# The Treatment of Warts

**Published:** 1899-01

**Authors:** 


### THE TREATMENT OF WARTS.

Louvel-Dulongpre advocates the following painless treatment, which also has the advantage of leaving no cicatrix: A concentrated solution of bichromate of potash in boiling water is prepared by gradually adding to the latter enough of the salt to make a saturated solution. On cooling, a certain quantity of the salt will again be precipitated. The supernatant fluid is to be applied once a day by means of a brush.—*Medicale Neuigkeiten*

